# Identifying Disengaged Responding in Multiple-Choice Items: Extending a Latent Class Item Response Model With Novel Process Data Indicators

**DOI:** 10.1177/00131644231169211

**Published:** 2023-04-29

**Authors:** Jana Welling, Timo Gnambs, Claus H. Carstensen

**Affiliations:** 1 Leibniz Institute for Educational Trajectories, Bamberg, Germany; 2 Otto-Friedrich University Bamberg, Germany

**Keywords:** disengaged responding, rapid guessing, process data, item response theory, computer-based assessments, multiple-choice items

## Abstract

Disengaged responding poses a severe threat to the validity of educational large-scale assessments, because item responses from unmotivated test-takers do not reflect their actual ability. Existing identification approaches rely primarily on item response times, which bears the risk of misclassifying fast engaged or slow disengaged responses. Process data with its rich pool of additional information on the test-taking process could thus be used to improve existing identification approaches. In this study, three process data variables—text reread, item revisit, and answer change—were introduced as potential indicators of response engagement for multiple-choice items in a reading comprehension test. An extended latent class item response model for disengaged responding was developed by including the three new indicators as additional predictors of response engagement. In a sample of 1,932 German university students, the extended model indicated a better model fit than the baseline model, which included item response time as only indicator of response engagement. In the extended model, both item response time and text reread were significant predictors of response engagement. However, graphical analyses revealed no systematic differences in the item and person parameter estimation or item response classification between the models. These results suggest only a marginal improvement of the identification of disengaged responding by the new indicators. Implications of these results for future research on disengaged responding with process data are discussed.

Educational large-scale assessments such as the *Program for International Student Assessment* (PISA; Schleicher, 2019) or the German *National Educational Panel Study* (NEPS; Blossfeld & Roßbach, 2019) administer cognitive tests to provide high-quality data on various domain-specific competences and their development over time. Still, a potential weakness of these studies is that they are low-stakes assessments in which the test results do not have direct consequences for the test-takers. Test-takers might thus lack the motivation to engage properly in the test and, consequently, respond to the items without investing their best effort, knowledge, and ability, a behavior defined as disengaged responding (Wise & DeMars, 2005). As disengaged responses do not reflect the ability of the test-takers, they pose a severe threat to the validity of the assessment (Wise, 2017).

Although various methods exist to identify disengaged responding, these typically rely either on item responses, item response times, or a combination of both (e.g., Pokropek, 2016; Ulitzsch et al., 2020; Wang & Xu, 2015). However, this practice bears the risk of misclassifications if fast engaged responses are mistaken for disengagement or slow disengaged responses are erroneously classified as engaged responses. Such misclassifications can in turn introduce bias into the estimation of item and person parameters. Therefore, additional indicators of response engagement might improve existing approaches by classifying item responses more accurately as engaged or disengaged. One potential source of information on test-taking behavior that has been increasingly available with the advent of computer-based assessments is process data, which provides a rich array of easily accessible information on the test-taking process. Few studies have yet investigated the potential of process data beyond item response times for the identification of disengaged responding (Ivanova et al., 2020; Lundgren & Eklöf, 2020; Patel et al., 2021; Sahin & Colvin, 2020). So far, available studies either concentrated on response engagement in interactive items or were rather exploratory. However, most competence tests in educational large-scale assessments are still based on traditional item formats such as multiple-choice questions. Furthermore, to evaluate the actual benefit of new indicators, it is essential to compare novel identification methods with already existing ones.

The aims of this study are thus (a) to derive novel indicators of response engagement from process data in a reading comprehension test, (b) to incorporate these indicators into an existing latent class item response model based solely on item response times, and (c) to evaluate the incremental value of the new indicators. After providing a brief overview of existing methods for the identification of disengaged responding, we will introduce the dependent latent class item response model (DLC-IRT) originally proposed by Pokropek (2016) and advanced by Nagy and Ulitzsch (2022). Subsequently, we will present the novel process data indicators of response engagement and derive an extended version of the DLC-IRT model. In the end, we will evaluate the new indicators by implementing both the original and the extended model in a sample of German university students as part of the NEPS.

## Types of Test-Taking Behavior

Schnipke and Scrams (1997) assumed that test-taking behavior can be distinguished into solution behavior and rapid guessing behavior. While solution behavior describes a state of engagement in which test-takers invest their best effort, knowledge, and ability into the response of an item, rapid guessing describes a state of disengagement in which test-takers respond to an item fast and without regard for its actual content. Although most previous research focused on rapid guessing, it is not the only possible form of disengaged responding. Slow disengaged responses, perfunctory answers to open format items, and item omissions can also be a consequence of disengagement in the test-taking process (Wise et al., 2020).

Numerous studies demonstrated that disengaged responding is a serious concern in educational large-scale assessments, with estimated prevalence rates up to 28%, depending on various aspects such as the content domain, setting, assessment mode, test-taker characteristics, country, or detection method (Goldhammer et al., 2017; Kroehne et al., 2020; Nagy & Ulitzsch, 2022; Rios et al., 2022). Disengaged responding manifests itself on the item-by-person level with engagement rates varying both between examinees and items (Ulitzsch et al., 2020). Consequently, disengaged responding is related to both person (e.g., ability, Wise et al., 2020) and item characteristics (e.g., item position, Lee & Jia, 2014). However, even though disengaged responding might be related to the ability of the test-taker or the item difficulty, disengaged responses do not reflect these properties in the same way as responses stemming from solution behavior. As a consequence, simply ignoring disengaged responding can lead to biased item and person parameter estimates (Jin et al., 2022; Rios et al., 2017). Furthermore, it can also influence conclusions and decisions on other aspects such as differential item functioning (DeMars & Wise, 2010) or the speed-ability relation (Deribo et al., 2022). These findings emphasize the necessity to identify and account for disengaged responding when using data from large-scale assessments to address substantial research questions.

## Identification of Disengaged Responding

Previous research has mainly focused on the identification of rapid guessing, a form of disengaged responding characterized by unrealistic short item response times (e.g., Wise, 2017). Identification methods can be generally divided into three different kinds of approaches: misfit-statistic-based, threshold-based, and model-based approaches. Misfit-statistic-based methods detect aberrant behavior by analyzing person-misfit statistics to identify extreme response patterns on the person level (e.g., Marianti et al., 2014). However, these methods fall short of identifying disengaged responding also on the item level. In contrast, threshold-based methods classify each item response as either engaged or disengaged (e.g., Goldhammer et al., 2017). If the respective item response time is shorter than a previously specified threshold, it is classified as disengaged. Disengaged responses are consequently filtered from the data set (e.g., Lee & Jia, 2014; Wise & DeMars, 2006). However, this approach requires setting either a common threshold for all items, which does not reflect item-specific time demands on the test-takers, or separate thresholds for each item, which may result in a cumbersome procedure especially for long tests. Since filtering responses classified as disengaged is based on the assumption that disengaged responding and ability are not related, ability estimates might be further biased if this assumption is violated.

Model-based methods simultaneously estimate ability and response engagement in a joint model (Pokropek, 2016; Ulitzsch et al., 2020; Wang & Xu, 2015). These mixture item response theory (IRT) models define response engagement as a latent class variable, assuming a mixed distribution for the probability of a correct response. In the solution behavior class, the probability follows a customary IRT model, whereas in the disengaged responding class, the probability of a correct response is independent of the test-taker’s ability and item difficulty. Model-based approaches differ in the way they incorporate item response times into the model (Nagy & Ulitzsch, 2022). Independent latent class IRT models assume a mixed distribution for item response time dependent on latent class membership and thus treat response engagement as an independent variable determining item response times (Ulitzsch et al., 2020; Wang & Xu, 2015). In contrast, DLC-IRT models incorporate item response times as a predictor of latent class membership into the model and thus treat response engagement as a dependent variable (Pokropek, 2016). Nagy and Ulitzsch (2022) provided a common multilevel framework for both approaches and illustrated with empirical data that both model types can indeed improve traditional IRT models ignoring disengaged responding.

A great advantage of the DLC-IRT model is the flexibility to extend the model by adding new predictors in the regression of latent class membership. Furthermore, it is less computationally extensive and thus rather forgiving when adding new parameters to the model. Due to these reasons, the DLC-IRT model is used in this study as the baseline model.

## A Dependent Latent Class Item Response Model for Disengaged Responding

The DLC-IRT model was originally proposed by Pokropek (2016) and is based on the grade of membership model of Erosheva (2002), which allows mixing proportions on the item-by-person level. The DLC-IRT model defines a latent class variable $C_{\mathrm{ij}}$ for person i and item j with the two latent classes *solution behavior* ( $C_{\mathrm{ij}}=1$ ) and *disengaged responding* ( $C_{\mathrm{ij}}=0$ ). The latent class is defined on the item-by-person level, allowing the proportions of the latent classes to vary between all item-by-person encounters. The probability of a correct response $P(Y_{\mathrm{ij}}=1)$ for person i on item j follows a mixed distribution and depends on the latent class. In the solution behavior class, the probability of a correct answer is defined by any IRT model. In this study, we define the probability as following a Rasch model (Rasch, 1960):

(1) $$P\left(Y_{ij}=1|C_{ij}=1\right)=\frac{\exp(\theta_{i}-\beta_{j})}{1+\exp(\theta_{i}-\beta_{j})},$$

where $Y_{\mathrm{ij}}$ denotes the item response for person i on item j . The parameter $\theta_{i}$ defines the ability of person i , and $\beta_{j}$ the item difficulty of item j . The probability of a correct response thus depends both on the ability of the examinee and the difficulty of the item. In the disengaged responding class, however, the probability of a correct response is assumed to be independent of both person and item characteristics and is hence defined by an item-specific constant $z_{j}$ :

(2) $$P\left(Y_{\mathrm{ij}}=1|C_{\mathrm{ij}}=0\right)=z_{j},$$

The size of $z_{j}$ depends on the item type. In constructed response items, the probability of a correct response for disengaged responding approximates zero. However, in multiple-choice questions, the test-takers can guess the items correctly even without investing any effort into the response. The probability of a correct response in the disengaged responding class should thus equal the probability of a random guess, which depends on the number of response options $M_{j}$ for item j . For multiple-choice questions with only one correct response option, we define $z_{j}$ as:

(3) $$z_{j}=\frac{1}{M_{j}}.$$

Overall, the probability of a correct response for person i on item j is thus defined as:

(4) $$P\left(Y_{\mathrm{ij}}=1\right)=\pi_{\mathrm{ij}}\frac{\exp\left(\theta_{i}-\beta_{j}\right)}{1+\exp\left(\theta_{i}-\beta_{j}\right)}+(1-\pi_{\mathrm{ij}})\;z_{j},$$

where $\pi_{\mathrm{ij}}$ defines the probability of belonging to the solution behavior class.

The probability of latent class membership for each item-by-person encounter is regressed on the response engagement indicators. In the model by Pokropek (2016), the only predictor in the logistic regression is the log-transformed item response time $l_{\mathrm{ij}}$ :

(5) $$P\left(C_{\mathrm{ij}}=1\right)=\frac{\exp(\alpha +\gamma l_{\mathrm{ij}})}{1+\exp(\alpha +\gamma l_{\mathrm{ij}})},$$

where α defines the general intercept and γ the regression coefficient for the predictor log-transformed item response time. This model assumes that item response times indicate disengaged responding equally well for all items, represented by the general intercept that can be understood as a general item response time threshold. However, the average time test-takers need to spend on the completion of an item might differ between items. Nagy and Ulitzsch (2022) thus proposed to replace the general intercept in Equation 5 with item-specific intercepts for J items, incorporating the varying time demands into the model:

(6) $$P\left(C_{\mathrm{ij}}=1\right)=\frac{\exp\left(\sum_{j=1}^{J}\alpha_{j}d_{j}+\;\gamma l_{\mathrm{ij}}\right)}{1+\exp\left(\sum_{j=1}^{J}\alpha_{j}d_{j}+\;\gamma l_{\mathrm{ij}}\right)},$$

where $\alpha_{j}$ defines the item-specific intercept and $d_{j}$ a dummy variable indicating whether the current item is item j . As the model is defined on one level only, the authors call it the dependent latent class single level item response theory (DLC-SL-IRT) model. As Equations 5 and 6 assume that persons do not differ regarding their response speed, it is also assumed that individual test-taking speed and ability are not correlated, contradicting several studies that suggest the opposite (e.g., Goldhammer et al., 2016; Lee & Jia, 2014; Nagy & Ulitzsch, 2022). Therefore, Nagy and Ulitzsch (2022) proposed a second extension of Pokropek’s model by adding the random effect $\psi_{i}$ to the logistic regression of latent class membership:

(7) $$P\left(C_{\mathrm{ij}}=1\right)=\frac{\exp\left(\sum_{j=1}^{J}\alpha_{j}d_{j}+\;\gamma l_{\mathrm{ij}}+\psi_{i}\right)}{1+\exp\left(\sum_{j=1}^{J}\alpha_{j}d_{j}+\;\gamma l_{\mathrm{ij}}+\psi_{i}\right)}.$$

The random effect $\psi_{i}$ reflects between-individual differences in response time thresholds. It is normally distributed with zero mean and the variance $\sigma_{\psi }^{2}$ . In this model, $\psi_{i}$ is allowed to covary with the individual ability $\theta_{i}$ . As the random effect introduces a second level to the logistic regression, the authors call it a dependent latent class two-level IRT (DLC-TL-IRT) model. In the empirical sample studied by Nagy and Ulitzsch (2022), both the DLC-SL-IRT and the DLC-TL-IRT model fitted the data better than a simple IRT model ignoring disengaged responding. Moreover, the DLC-TL-IRT model exhibited a better model fit than the DLC-SL-IRT model. However, the authors did not compare the extended models to the original DLC-IRT model proposed by Pokropek (2016).

## Enhancing the Identification of Disengaged Responses with Process Data

Similar to other identification approaches, the DLC-IRT model solely relies on item responses and item response times as indicators of response engagement, risking misclassifications of slow disengaged or fast engaged responses. Additional indicators of response engagement might thus improve the DLC-IRT model. As process data offers a rich pool of additional information on the test-taking process, it can be a major source of novel indicators.

As of yet, only few studies have investigated the potential of process data for the identification of disengaged responding. In Lundgren and Eklöf (2020), clusters of process data representing test-taking strategy and effort predicted self-reported effort and test performance in an interactive problem-solving task. Sahin and Colvin (2020) showed that the number and type of actions could in some cases slightly improve the response time thresholds in interactive tasks. Ivanova and colleagues (2020) found that the number of actions in constructed response items was related to test performance, self-reported test-taking effort, and item position. Finally, Patel and colleagues (2021) used a machine learning approach to predict test-taking efficiency in a test of mixed item types. Besides measures related to test or item response time, the number of certain actions (e.g., answer changes and navigation button use) were identified as important features of test efficiency.

These findings indicate that process data beyond response times might indeed possess valuable information for the identification of disengaged responding. However, the aforementioned studies focused mostly on interactive or constructed response tasks. Although these item types are becoming increasingly popular in educational large-scale assessments, most competence tests are still based on traditional item formats such as multiple-choice questions. Furthermore, most existing studies were rather exploratory. Therefore, this study further explores the relationship of process data from multiple-choice items of a reading competence test with test-taking engagement. We aim to derive novel indicators of response engagement and incorporate them into a model-based approach of disengagement. In doing so, we hope to refine existing response-time based approaches to facilitate interpretations of disengagement and its correlates.

## The Current Study

### Development of Novel Indicators of Response Engagement

The novel indicators of response engagement were derived based on theoretical considerations. Each indicator is defined on the item-by-person level, so they can differ between all item-by-person encounters. Initially, all possible kinds of interactions with multiple-choice items in reading tasks were identified. A reading test usually consists of several tasks with a text at the beginning and subsequent items referring to the text. The text and each item are each presented on single pages of the test. Therefore, the test-taker can interact with the task by navigating through the pages, either between a text and an item page or between two item pages. In multiple-choice items, test-takers can choose between several response options with usually only one response option being correct. The test-taker thus can additionally interact with the items by selecting and changing response options. Based on these considerations, we derived three potential indicators of response engagement.

First, the test-taker can choose to navigate back to the text while working on an item. Thus, we define the indicator *text reread* as the navigation from an item page back to the corresponding text page. When the test-taker navigates back to the text, it can be assumed that she or he has actually read the content of the item and now wants to collect more information to be able to respond to the item. The test-taker hence allocates resources to the item response, contradicting the definition of disengaged responding and thus indicating solution behavior.

Second, the test-taker might navigate to an item page she or he has already visited before. We thus define the indicator *item revisit* as the repeated navigation to an item page. The revisit of an item might occur for different reasons. The test-taker might (a) first scan the item and return to it later for the actual response, (b) return to a difficult item she or he was not able to solve before, (c) review an item she or he has already responded to, or (d) navigate aimlessly through the item pages. In the first three cases, the test-taker shows her or his will to properly engage with the item. However, in the last case, no conclusions regarding the test-taking behavior on single items can be drawn, as a random navigation behavior might occur either in between engaged item responses or due to a state of disengagement. As we do not expect this behavior to occur frequently, we assume that the revisit of an item nevertheless indicates solution behavior.

Third, a test-taker might change an already provided response to an item. Thus, we define the indicator *answer change* as changing the previously selected response option to another one. An answer change might occur for three reasons. The test-taker might (a) review the item and subsequently change their belief about the correct response option, (b) correct an accidentally provided response, or (c) select accidentally another response option. In the first two cases, the test-taker has apparently read the item and allocated effort and knowledge into the response, indicating solution behavior. In the third case, however, no strong assumptions regarding the test-taking behavior can be made and thus can be seen as noise in the data.

In total, we identified three novel indicators that can be derived from process data in multiple-choice items of a reading test: text reread, item revisit, and answer change. All three indicators are expected to indicate solution behavior and thus contradict disengaged responding.

### Research Questions and Hypotheses

To evaluate the new indicators of response engagement, they were incorporated into an extended version of the DLC-IRT model and compared with a baseline model that only acknowledged item response times. As a potential baseline model, we considered the original DLC-IRT (Pokropek, 2016), the DLC-SL-IRT, and the DLC-TL-IRT model (Nagy & Ulitzsch, 2022). The extended DLC-IRT model included the new indicators as additional predictors in the logistic regression of the latent class membership as defined in Equations 5 to 7. Note that the interpretation of the item-specific intercepts $d_{j}$ and the random effect $\psi_{i}$ alter when additional engagement indicators are added to the regression. In this case, these parameters rather represent item-specific engagement difficulties and an individual propensity to show solution behavior, respectively.

We expected the new indicators to enhance the baseline DLC-IRT models by improving the classification of response engagement. In the new model, we assumed item response time to be a significant predictor of latent class membership, replicating the findings of Pokropek (2016) and Nagy and Ulitzsch (2022). However, we did not expect all auxiliary indicators to be equally informative. Specifically, we expected the indicator *item revisit* to be at least partly redundant with item response times, as revisiting the item page simultaneously extends the item response time by the time of the additional visit. Instead, we expected the indicators *answer change* and *text reread* to be more informative and thus the only significant new predictors of latent class membership. We hence propose the following hypotheses:

1. The extended DLC-IRT model describes the observed responses better than the best baseline model.
2. The indicators *item response time*, *answer change*, and *text reread* predict latent class membership, so that responses are more likely to be classified as solution behavior when test-takers need more time for the response, change their answer, or reread the text.
3. The indicator *item revisit* does not predict latent class membership over and above item response time.

The relationship between response engagement and its indicators might not solely be linear. For example, the indicators might only improve the classification of response engagement when response times are long enough that the test-takers could have read and processed the item. To allow also for nonlinear effects, we also propose a second extension of the DLC-IRT model by further introducing interactions between the indicators as additional predictors in the logistic regression. As this extension is exploratory, we include all six possible interaction terms in the regression but have no specific a priori hypotheses.

## Method

### Sample

This study used a subsample from the student cohort in the NEPS, which assesses competence development and educational trajectories across the life span (Blossfeld & Roßbach, 2019). In Wave 12, reading competence was assessed in a quasi-experimental design, with the sample being split between a computer-based, proctored and a web-based, unproctored test setting. To avoid setting or mode effects, only the data of the web-based test was used. The 1,932 participants (59.4% female) were aged from 23 to 59 years (*M* = 28.06, *SD* = 3.59). The majority identified German as their native language (94.9%) and was employed at the time of assessment (85.8%).

### Instruments and Measures

#### Test of Reading Comprehension

The test of reading comprehension in German was developed specifically for the NEPS (Gehrer et al., 2012) and included five reading tasks. Each task consisted of a short text and a respective item set referring to this text. The test comprised a total of 21 items with different response formats. However, for the present analyses, only the 14 simple multiple-choice items were used. Each of these items included four response options with one correct solution and three distractors.

After a standardized instruction, the main part of the test allowed participants to navigate within each task, but not between different tasks. Each text and each item was presented on different pages (only very long texts were presented on two consecutive pages). Navigation between different pages within a task was possible by either selecting the forward or backward button on each page or by clicking on a specific page name in the navigation panel. Thus, all text and item pages could be visited several times. The tasks could be finished by selecting the forward button on the last page of the task. However, participants were then asked if they definitely wanted to leave the task, as they were unable to return to it later. Response options of the multiple-choice items could be selected by a mouse click on the respective radio button.

#### Indicators From Process Data

Process data were continuously logged while the participants took the test. Mouse clicks, the selection of buttons, and the use of the navigation panel were each logged with a corresponding time stamp. This study includes only navigation and response option selection events (*N* = 85,848). Preliminary analyses revealed that several participants navigated through the test using only the forward and backward buttons. This resulted in numerous navigation events that did not reflect the participants’ intention to *stay* on the selected test page but rather occurred as intermediate navigation steps. These irrelevant navigation events prevent an accurate assessment of some of the response engagement indicators and were thus filtered from the data, resulting in a new sample of 71,070 log events. More information on the implementation of the navigation filter and the exclusion criteria can be found in the Supplementary Material.

The four disengaged responding indicators were each defined on the item level. *Item response time* was defined as the time the participant spent in total on the respective item page. To determine the item response times, the time interval for each page visit was calculated. The start of the time interval was defined as the click on a navigation button that led to the respective item page and the end was defined as the click on a navigation button to leave the respective item page. If a participant visited the page several times, the item response time was defined as the sum of all page visit time intervals. As the distribution was heavily right-skewed, the log response time was used for the analyses.

An *answer change* was registered each time when a participant had already selected one response option before and, subsequently, selected another response option within the same item. A *text reread* was defined as navigating from the respective item page to a text page. An *item revisit* was registered when three conditions were met. First, the participant navigated to the respective item page. Second, it was not the first visit to this item page. Third, between the last and the current visit of the item, at least one other item was visited (to distinguish text rereads from item revisits). The frequency distributions of the three new indicators are given in Table 1. As all indicators were severely skewed, they were dichotomized for the analyses (1 = occurred at least once, 0 = did not occur).

**Table 1.** Frequency Distributions for New Indicators of Response Engagement.

Indicator	Frequency				
	0	1	2	3	>3
Answer change	21,642	2,224	1,017	182	63
Text reread	16,875	4,933	2,024	784	512
Item revisit	23,440	1,521	145	17	9

### Statistical Analyses

Data preparations, descriptive analyses, and visualizations were conducted in R (Version 4.0.3/4.2.2; R Core Team, 2021), whereas the IRT models were estimated in *Mplus* (Version 8.4; Muthén & Muthén, 1998-2017). Although the scored test data are provided at NEPS Network (2022), the process data cannot be made publicly available due to legal restrictions. However, the computer code and analysis results are provided in the Supplemental Material.

We fitted six different IRT models to the data, beginning with a Rasch model that ignored disengaged responding. Then, the original DLC-IRT model, the DLC-SL-IRT model, and the DLC-TL-IRT model as defined in Equations 1 to 7 with item response time as the only indicator of response engagement were estimated as potential baseline models. The models were compared using Akaike’s Information Criteria (AIC; Akaike, 1974), Bayesian Information Criteria (BIC; Schwarz, 1978), and sample size adjusted BIC (aBIC), as well as log-likelihood ratio tests. The best fitting model was used as the baseline model for further analyses.

As fifth model, the baseline model was extended by adding the three new dichotomized response engagement indicators as additional predictors in the regression of latent class membership. In the sixth model, all possible interaction terms of the four indicators were added as further predictors to the same regression. Both extended models were then compared with the Rasch and baseline model using information criteria and deviance tests.

All models were implemented as defined in Equations 1 to 7 by adapting the Mplus syntax provided in the Supplementary Material of Nagy and Ulitzsch (2022). The probability of a correct response followed a Rasch model in the solution behavior class, but was set to chance level (25%) for all items in the disengaged responding class. When the item response time didn’t exceed zero seconds (*n* = 1,934), which only occurred in combination with a missing item response, all four process data indicators were defined as missing. As small numbers of missing responses do not introduce severe parameter bias in IRT models (Rose et al., 2010), missing data were ignored in the analyses. Starting values were set to 80/8 and increased to 250/25 when the best loglikelihood had not been replicated. All models were estimated using the marginal maximum likelihood procedure. Entropy as an index of classification accuracy (Asparouhov & Muthén, 2018) is reported for each model. Because previous research suggests that the probability of correct guesses may indeed exceed what should be expected by chance alone (e.g., Ulitzsch et al., 2021), we re-estimated in a sensitivity analysis the best fitting model with equal, but not fixed item difficulties in the disengaged responding class, and report the results in the Supplementary Material.

## Results

### Descriptive Results

The mean item response times (ranging from 17.35 to 36.24 seconds, *Mdn* = 27.77) and the proportions of participants who changed their answer at least once (ranging from 8.5% to 20.7%, *Mdn* = 13.5%) differed only slightly between items (see Table 2). We observed more pronounced differences for the proportion of correct answers (ranging from 29.8% to 83.8%, *Mdn* = 52.9%) and the percentage of participants who reread the text (ranging from 5.7% to 68.2%, *Mdn* = 35.8%) or revisited the item at least once (ranging from 1.0% to 14.0%, *Mdn* = 5.3%). Although all four indicators correlated moderately with each other (see Table 3), we did not observe substantial multicollinearity. The largest correlation resulted between the log item response times and the dichotomized text rereads (*r* = .30, *p* < .001).

**Table 2.** Descriptive Results Per Item.

Item	Item-by-person encounters	Response time	Item responses	Answer change	Text reread	Page revisit	Probability to belong to the solution behavior class
	*N*	*M* (*SD*)	Percentage correct	At least once	At least once	At least once	
1	1,930	30.17 (28.09)	70.7%	15.1%	68.2%	14.0%	89.8%
2	1,928	36.24 (18.58)	64.8%	18.6%	49.5%	11.9%	86.7%
3	1,928	27.87 (15.77)	32.9%	12.4%	55.9%	5.0%	43.5%
4	1,898	26.06 (18.27)	83.8%	20.7%	11.0%	4.4%	97.8%
5	1,900	21.67 (12.85)	49.9%	11.7%	5.7%	2.9%	95.9%
6	1,872	27.67 (14.62)	54.1%	14.6%	40.3%	10.4%	96.9%
7	1,867	21.03 (12.74)	50.0%	15.4%	7.3%	9.6%	67.3%
8	1,856	29.85 (17.64)	38.2%	16.9%	40.0%	11.7%	96.4%
9	1,849	33.03 (17.53)	71.2%	8.5%	8.3%	9.3%	96.5%
10	1,808	27.36 (13.68)	39.5%	12.1%	36.1%	1.4%	96.1%
11	1,733	32.55 (27.07)	51.6%	15.1%	35.5%	1.2%	68.9%
12	1,617	32.44 (17.80)	63.6%	9.7%	18.7%	1.0%	94.9%
13	1,464	22.34 (12.32)	29.8%	11.7%	28.8%	5.6%	93.7%
14	1,413	17.35 (12.23)	69.9%	9.8%	57.1%	3.3%	94.8%

**Table 3.** Correlations Between Indicators of Response Engagement and Item Responses.

Variable	*M*	*SD*	1	2	3	4
1. Item response	0.55	0.50				
2. Response time	3.16	0.59	.08***			
3. Answer change	0.14	0.35	–.01	.23***		
4. Text reread	0.33	0.47	.01*	.31***	.12***	
5. Page revisit	0.07	0.25	.00	.18***	.16***	.10**

*Note.* Presented are Pearson correlation coefficients. Response time refers to logarithmized response time.

* *p* < .05. **p < .01. ***p < .001.

### Baseline Models for Disengaged Responding

In the first step, the baseline model for disengaged responding was identified. To this end, we compared the ordinary Rasch model that does not acknowledge disengaged responding, the DLC-IRT with a common intercept for all items, the DLC-SL-IRT with item-specific intercepts, and the DLC-TL-IRT with an individual threshold for response engagement (see Table 4). Importantly, the three DLC models only included item response time as a predictor of class membership. Because the DLC-SL-IRT model was empirically underidentified when considering unique intercepts for all items, item-specific intercepts were identified using a sequential process. First, a model was estimated for each item that included a general intercept and the respective item-specific intercept. Then, this model was compared with the DLC-IRT model without the item-specific intercept. Only those item-specific intercepts were retained for which model comparisons provided a better fit to the data. In the end, the DLC-SL-IRT model comprised a general intercept and item-specific intercepts for Items 1, 2, 3, 7, 11, and 14. The model comparisons showed the best fit for the DLC-TL-IRT model with six item-specific intercepts and a random effect for the individual speed. This model was therefore used as the baseline model for the subsequent analyses.

**Table 4.** Model Comparisons for Item Response Models.

Number	Model	*n_par_*	Information criteria			Entropy	χ^2^-test		
			AIC	BIC	aBIC		ΔDev	*df*	*p*
1.	Rasch	15	31,106	31,106	31,180				
	Baseline models								
2.	DLC-IRT	17	30,944	31,082	31,028	0.98	166.09	2	<.001
3.	DLC-SL-IRT	23	30,730	30,917	30,844	0.93	225.81	6	<.001
4.	DLC-TL-IRT	25	30,697	30,900	30,821	0.87	37.35	2	<.001
	Extended models								
5.	DLC-TL-IRT + new indicators	28	30,678	30,906	30,817	0.87	24.68	3	<.001
6.	DLC-TL-IRT + new indicators + interactions	34	30,659	30,935	30,827	0.86	31.68	6	<.001

*Note*. aBIC = sample-size adjusted BIC; ΔDev = difference in deviance; DLC-IRT = dependent latent class item response theory; DLC-SL-IRT = single-level DLC-IRT; DLC-TL-IRT = two-level DLC-IRT.

*n_par_* = number of parameters; AIC = Akaike’s information criterion (Akaike, 1974); BIC = Bayesian information criterion (Schwarz, 1978).

### Extended Models for Disengaged Responding

As compared with the baseline model, the loglikelihood ratio test and the AIC indicated a better fit of the extended DLC-IRT model that included the main effects of the new indicators of response engagement and also the extended DLC-IRT model that additionally included the respective interactions (see Table 4). Moreover, both criteria slightly favored the most complex model also including interactions. In contrast, the aBIC slightly favored the extended model without interactions, while the BIC slightly favored the baseline model over both extended models. Given the mixed results, we selected the simpler model without interactions for the subsequent analyses (=*extended model*), thus supporting Hypothesis 1.

The entropy of the extended model was 0.87 and the average probability for item responses to be classified as solution behavior based on the posterior probability distribution was 86.7%. However, as can be seen in Table 2, this probability differed strongly between items. Item 3 exhibited an average probability of 43.5% and Item 4 exhibited an average probability of 97.8% for item responses to belong to the solution behavior class. More items were responded correctly (60.2%) and participants needed longer to respond to an item (*M* = 29.67 seconds, *SD* = 17.93) in the solution behavior class than in the disengaged responding class (19.4%, *M* = 14.15 seconds, *SD* = 8.07). Furthermore, participants more often changed their answer (14.8%), reread the text (33.7%) and revisited the item (7.2%) in the solution behavior class than in the disengaged responding class (7.8%, 26.0%, and 3.2%, respectively).

The item-specific intercepts for Items 1, 2, 3, 7, and 11 were significant (*p* < .05), with a smaller probability to be classified into the solution behavior class for these items (see Table 5). The variance of the random effect for the individual speed was significant (*Var* = 7.26, *p* = .049) and correlated perfectly with the latent reading ability (*r* = −1.00, *p* < .001). However, this result should be interpreted only carefully as the standard error of the variance of the random effect was rather large (*SE* = 3.69). Item response time and text reread were significant predictors of latent class membership. The longer a participant needed for an item response, the higher was the probability that this item response belonged to the solution behavior class (*B* = 11.26, *p* < .001). In contrast to our expectations, rereading the text while working on an item was associated with a higher probability of being *disengaged* (*B* = −2.60, *p* = .001). The other indicators were no significant predictors of latent class membership. Hypothesis 2 could thus only be partly confirmed for the predictor item response time, while Hypothesis 3 was supported by the results.

**Table 5.** Results of Hierarchical Linear Modeling.

Parameter	Estimate	*SE*	*t*	*P*
Within level				
General intercept	–21.52	4.65	–4.63	<.001
Intercept Item 1	–6.34	1.57	–4.04	<.001
Intercept Item 2	–9.83	2.16	–4.56	<.001
Intercept Item 3	–13.93	2.74	–5.08	<.001
Intercept Item 7	–8.43	1.78	–4.74	<.001
Intercept Item 11	–13.17	2.76	–4.77	<.001
Intercept Item 14	3.92	2.21	1.78	.076
Item response time	11.26	2.28	4.94	<.001
Answer change	–0.76	0.79	–0.96	.339
Text reread	–2.60	0.79	–3.30	.001
Item revisit	–1.43	1.01	–1.42	.156
Between level^a^				
Variance of $\psi_{i}$	7.26	3.69	1.97	.049
Covariance between $\psi_{i}$ and $\theta_{i}$	2.69	0.68	3.94	<.001
Correlation between $\psi_{i}$ and $\theta_{i}$	1.00	0.01	68.94	<.001

*Note.* $\psi_{i}$ defines the individual threshold for response engagement and $\theta_{i}$ the individual reading proficiency of person i .

a The variance of $\theta_{i}$ was fixed to 1 for identification.

**Figure 1.**
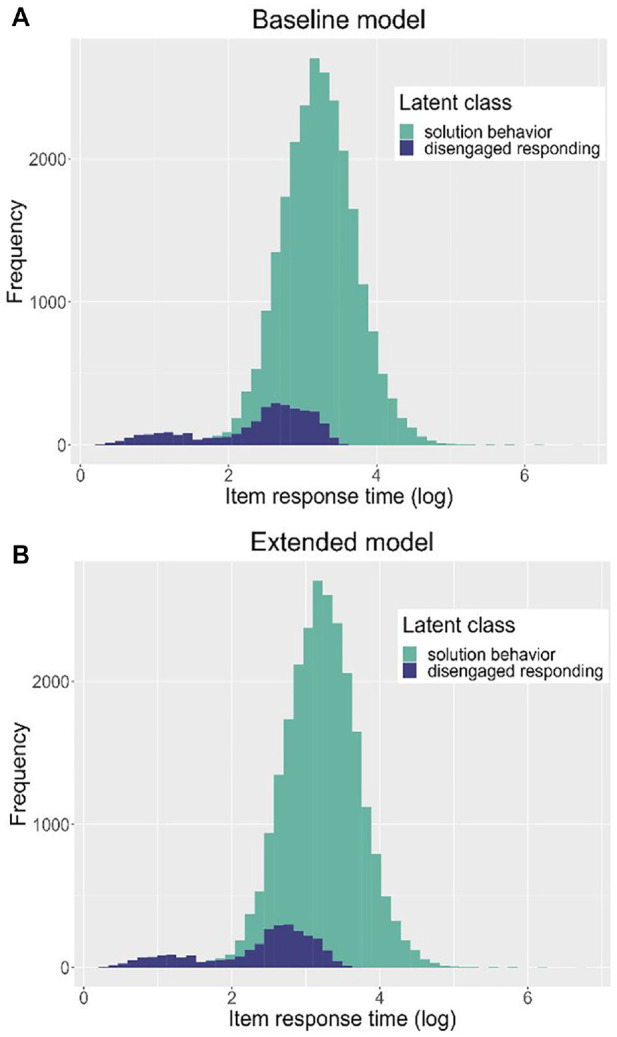
Distribution of Item Response Times by Latent Class and Response Engagement Model.

One of the assumptions of this study was that the new indicators of response engagement can improve the DLC-IRT model by reducing the error rates of the (dis)engagement classification, as slow disengaged or fast engaged responses might be misclassified when item response time is the only predictor of latent class. Figure 1 displays the distribution of item response times by latent class membership for both the baseline and the extended model. As the item response time distributions look basically the same for both models, the new indicators do not seem to substantially reduce the error rates of engagement classification.

**Figure 2.**
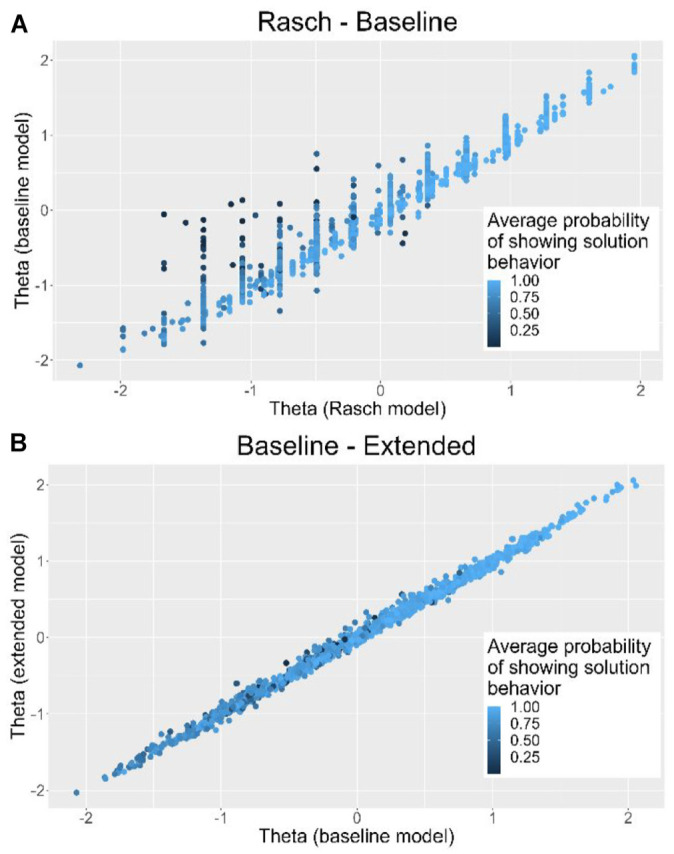
Comparison of Proficiency Estimates Between the Rasch, Baseline, and Extended Model.

**Figure 3.**
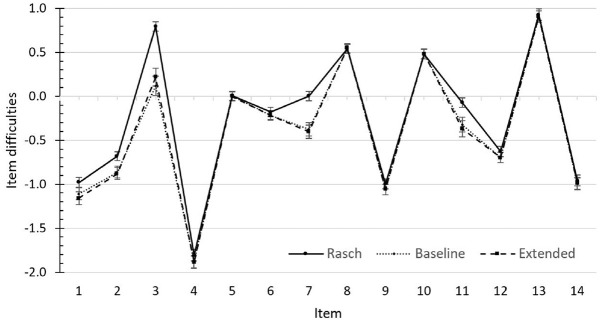
Comparison of Item Difficulties Between the Rasch, Baseline, and Extended Model.

A second assumption of this study was that the new indicators of response engagement can reduce the bias in person and item parameters by improving the prediction of latent class membership. As can be seen in Figure 2, the competence estimates were systematically underestimated in the Rasch model when participants tended to respond in a disengaged manner. However, the figure does not show any systematic difference in competence estimates between the baseline and the extended model. The item difficulties are displayed in the Supplementary Material. Figure 3 indicates that the Rasch model overestimated item difficulties for some of the items (notably the items for which item-specific intercepts were included in the model). However, no major changes in item difficulties between the baseline and the extended model could be observed. The new indicators of response engagement thus do not seem to substantially reduce bias in the person or item parameters.

## Discussion

Because disengaged responding poses a serious threat to the validity of educational large-scale assessments, various methods have been proposed to identify them and correct for their distorting influence. Most of these approaches rely solely on item responses and item response times (e.g., Pokropek, 2016; Ulitzsch et al., 2020; Wang & Xu, 2015), while ignoring potentially useful information on the test-taking process provided by additional process data. To further enhance our understanding of test-taking behavior, this study derived novel indicators of response engagement from process data and evaluated whether they improve model-based identification approaches of disengaged responding. In a sample of German university students, an application of the extended latent class item response model including the three new indicators as additional predictors of response engagement led to three central findings.

In line with Hypothesis 1, the extended DLC-TL IRT model that included the new indicators showed a better fit as compared with a model with response times alone, supporting the finding of previous studies that process data beyond response time might indeed provide valuable information on response engagement (Ivanova et al., 2020; Lundgren & Eklöf, 2020; Patel et al., 2021; Sahin & Colvin, 2020). However, a closer inspection of the results revealed that only item response times and text reread predicted response engagement while answer change and item revisit did not. Supporting Hypothesis 2, shorter item response times were associated with a greater likelihood of disengaged responding, thus replicating numerous previous studies on disengaged responding (e.g., Pokropek, 2016; Ulitzsch et al., 2020; Wang & Xu, 2015).). Similar to the findings of Patel and colleagues (2021), the result further indicates that also in the presence of other process data indicators, item response time stays a principal indicator of response engagement. Moreover, it suggests that item response time is a valid indicator of response engagement also in unproctored settings when test-takers can engage in other activities while working on the test or even choose to temporarily leave the test.

Over and above this effect, rereading the text while working on an item predicted the probability of disengaged responding. Although the direction of the effect was in contrast to our initial hypotheses, this might reflect a suppression effect. Text rereads were moderately correlated with item response times and thus the information gained from this indicator is partially redundant with the information gained from item response time. The descriptive results support this assumption, since the percentage of text rereads was lower for disengaged item responses than for engaged item responses. However, text rereads seem to further distinguish between disengaged responding and solution behavior after conditioning on the item response times. In this case, text rereads might help to identify disengaged test-takers that navigate back to the text page rather aimlessly instead of directly guessing the item at hand. Finally, the two remaining indicators, answer change and item revisit, showed no incremental contribution to the prediction of latent class membership. This is likely a consequence of the restricted range of these indicators because both behaviors were rarely observed and, thus, provided little information about the test-taking process. Furthermore, these indicators were once more redundant with the information gained from the item response times. Taken together, the results highlight that indicators derived from process data can inform about response engagement. But whether the information they provide is relevant beyond response times strongly depends on the specific indicator.

Despite the relevance of text rereads for the prediction of response engagement, we found no systematic bias in students’ reading proficiencies or item difficulty when not accounting for the additional indicators of response engagement. Furthermore, histograms of item response times by latent class revealed no visible classification differences between the models for both slow and fast item responses. These results contradict our assumption that the novel indicators might be able to improve the classification of (dis)engaged responses in cases where item response times do not suffice as the only indicator (e.g., fast engaged and slow disengaged responses).

### Accuracy of Response Engagement Classification

In the extended model, the probability of showing solution behavior varied drastically between items, with one item even exhibiting a probability of less than 50%. In other model-based approaches, however, prevalence rates per item do usually not fall below 76% (e.g., Nagy & Ulitzsch, 2022; Ulitzsch et al., 2021). Moreover, response options in the disengaged responding class were selected with differing frequency (rates ranged between 1% and 67%), instead of being equally distributed, as should be expected for random guesses. Frequently selected response options were mostly wrong response options, indicating rather strong distractors than random guessing. This assumption is supported by the fact that, averaged over all items, the probability of a correct response in the disengaged responding class was estimated to be less than chance level. Freeing the item difficulties of the disengaged responding class in the sensitivity analysis resulted in an even lower probability of a correct response for disengaged responses. Furthermore, graphical analyses revealed that also numerous slow responses were classified as being disengaged, both in the baseline and extended model. Finally, the individual threshold for response engagement exhibited a nearly perfect correlation with reading proficiency. Although response engagement and ability were repeatedly shown to be related, the size of the correlation is usually estimated to range between .25 and .70 (e.g., Goldhammer et al., 2016; Ulitzsch et al., 2021). This suggests rather limited evidence for individual differences in the threshold for response engagement in the present sample. After all, the respective random variance was only borderline significant. Consequently, the respective correlation should be interpreted only carefully.

These findings indicate that in the present response engagement model, more responses were classified as disengaged than should be expected when the assumption that disengaged responses are equal to random guesses were true. Thus, also responses that were provided with little or decreasing effort or engaged responses of low-ability test-takers might have been classified as disengaged. As rereading the text predicted disengaged responding, “disengaged” test-takers seem to have started solving an item and reread the text to find the correct answer, but might not have perceived the correct response in the end. The strong correlation between reading proficiency and response engagement further suggests that these two constructs were to some degree confounded in the dependent latent class item response model of this study.

Since these results were also found (though sometimes a bit extenuated) in the baseline model, they are not explainable by the model extension alone. In contrast to Nagy and Ulitzsch (2022), the present study implemented the response engagement model in multiple-choice questions instead of open-end questions. The DLC-TL-IRT model might thus have difficulties distinguishing between disengaged wrong guesses and engaged wrong answers when the success probability of disengaged responses does not approximate zero. However, also the setting, the test, and the sample differed between the two studies. Whether one of these factors might have influenced the outcome as well, can only be answered by future studies testing the applicability of the DLC-TL-IRT model in different conditions.

### Implications for Educational Large-Scale Assessments

The results emphasize the importance of item response time as an indicator of response engagement while estimating the incremental value of the selected additional process data indicators as rather weak. One possible explanation for this finding is that the vast majority of disengaged responding might indeed be rapid guessing (as suggested by Wise et al., 2020), rendering item response times a sufficient single indicator of response engagement. However, even if disengaged responding beyond rapid guessing did exist, the additional process data indicators might simply not provide enough information on the test-taking process to improve the classification of response engagement. Combining or rearranging the selected indicators (e.g., by clustering) or including other process data (e.g., mouse or eye movements) as potential indicators might improve the explanatory power. However, the results could also indicate that process data might generally possess only weak explanatory power for response engagement in multiple-choice items, as only little interaction between the test and the test-takers takes place. Future studies should thus systematically investigate the predictive value of different process data indicators for response engagement in multiple-choice items, while also taking into account other measures of disengagement (e.g., self-reports).

Our findings suggest that the DLC-TL-IRT model did not only classify random guesses as disengaged. We see two different explanations for this finding. First, item responses might not simply be classified as either engaged or disengaged, but some intermediate steps or continuum might exist which are confounded with the ability in the test domain. A. Pokropek (personal communication, July 14, 2022) therefore suggested adding a *slow guessing* latent class to the DLC-IRT model. This latent class includes all responses of test-takers who first try to solve the item but then give up, resulting in long item response times and interactions with the item (e.g., rereading the text). Second, the result might also indicate that the DLC-IRT model does not function in multiple-choice items as well as in open-end item formats. Future studies should thus further investigate the applicability of the different DLC-IRT models to tests with multiple-choice items and compare it to other existing identification methods of disengaged responding. In addition, it might be insightful to include a third response engagement class in the DLC-IRT models and compare it to models with only two latent classes.

### Strengths and Limitations

This study contributed to the literature on disengaged responding in several ways. On one hand, it is one of rather few attempts to evaluate the potential of process data beyond item response times for the identification of disengaged responding and, thus, deepens our understanding of influential test-taking behavior affecting outcomes of reading comprehension tasks. On the other hand, the study not only investigated the validity of a model-based approach for the identification and handling of disengaged responding, but also built on these prior developments to improve existing methods. Therefore, the results of this study can help to further minimize the threat to test validity introduced by disengaged responding.

Besides its considerable strengths, the study also possesses some limitations. First, for reasons of practicability, we chose a study with a rather homogeneous sample and a rather short test. Because previous studies showed that age, educational attainment, and country can be associated with disengaged responding (e.g., Goldhammer et al., 2016; Lindner et al., 2019), more representative samples might identify more disengaged responses and, thus, stronger effects for the novel indicators. Second, the extended model included only one random effect for all four response engagement indicators. By not accounting separately for individual differences in the indicators, the model ignores potential relationships between the indicators on the person-level (which do exist; e.g., Bezirhan et al., 2021). Future studies could handle this problem by including random slopes in the model. However, random slopes are computationally intensive and thus could lead to non-identified models. Third, item properties were not included in the present study as potential indicators of response engagement. Previous studies have shown that disengaged responding is indeed related to item parameters such as item position (Goldhammer et al., 2016) and item content (Rios & Soland, 2022). Fourth, the indicator item revisit might not have been very reliable. As outlined in the Supplementary Material, we filtered irrelevant navigation events to enable the determination of reliable indicators. However, the filter might not be perfect and thus result in falsely identified item revisits, obscuring potential relationships with other variables.

Future studies might thus include additional parameters to the DLC-IRT models, further explaining the complex relationships between the indicators and adding new explanatory variables. Other promising future directions of research could be to test the generalizability of the current findings by applying them to other item formats or other test domains. All three process data indicators can also be defined for other item formats such as complex multiple-choice tasks and text enrichment tasks, although the indicator answer change might have to be slightly altered. As test-takers interact more with the test items of other formats, the additional indicators might convey more information on the test-taking process in these cases. Similarly, the process data indicators can mostly be transferred to other test domains, such as mathematics or foreign language tests. Naturally, the indicator text reread only applies to tests with an initial text or task description and respective item sets.

## Conclusion

The advent of computer-based assessments offers with process data a wide array of new information on the test-taking process. Nonetheless, studies investigating the potential of process data for the identification of disengaged responding are still rare. To fill this gap, this study sought to identify new process data indicators of response engagement in multiple-choice items and determine their predictive value. Three new potential indicators were introduced: answer change, text reread, and item revisit. In an empirical study, a DLC-IRT model with item response time as the only predictor of response engagement was compared with a DLC-IRT model additionally including the new indicators. The results suggest that the incremental predictive value of the new indicators is weak, while short item response times stay an important indicator of disengaged responding. Counterintuitively, text rereads seem to be associated with disengaged responding for equal item response times. However, further findings also indicate that in the present study, the DLC-IRT model does not only identify random guesses as disengaged but also presumably engaged but wrong answers. Future studies shall investigate whether this generally applies to tests based on multiple-choice items. Overall, this study contributes to the literature on disengaged responding by enhancing our understanding of test-taking behavior and further improving existing identification methods, thus minimizing the threat to test validity introduced by disengaged responding in the first place.

## Supplemental Material

sj-docx-1-epm-10.1177_00131644231169211 – Supplemental material for Identifying Disengaged Responding in Multiple-Choice Items: Extending a Latent Class Item Response Model With Novel Process Data Indicators Supplemental material, sj-docx-1-epm-10.1177_00131644231169211 for Identifying Disengaged Responding in Multiple-Choice Items: Extending a Latent Class Item Response Model With Novel Process Data Indicators by Jana Welling, Timo Gnambs and Claus H. Carstensen in Educational and Psychological Measurement
