# Supplementary material for: Identifying Disengaged Responding in Multiple-Choice Items: Extending a Latent Class Item Response Model With Novel Process Data Indicators
Source: Educ Psychol Meas. 2023 Apr 29;84(2):314–39. doi: 10.1177/00131644231169211 (PMC11185098; doi:10.1177/00131644231169211)
Supplement: sj-docx-1-epm-10.1177_00131644231169211 – Supplemental material for Identifying Disengaged Responding in Multiple-Choice Items: Extending a Latent Class Item Response Model With Novel Process Data Indicators [file sj-docx-1-epm-10.1177_00131644231169211.docx]

**Filtering irrelevant navigation events**

For the generation of the four indicators it is crucial to include only such navigation events that occur because the participant intends to *stay* on the selected page. However, some participants chose to navigate solely through the forward and back buttons, resulting in many visits of pages the participants in fact did not intend to stay on (e.g., if a participant works on item 3 and chooses to reread the text, the navigation pattern would be item 3 – item 2 – item 1 – text – item 1 – item 2 – item 3, even though the intended navigation is item 3 – text – item 3). Furthermore, some navigation events might occur completely unintended (e.g., a participant clicks on the wrong page in the navigation panel). Thus, navigation events that fulfilled the following conditions were excluded from analysis: a) the visited page is an item page, b) the page visit time is shorter than two seconds, c) the participant did not interact in any way with the item while being on the page, and d) the previous and the next page are not both a text page (in order to include “back and forth” jumping between an item and a text page). A comparison of the number of registered indicator events with and without filter and a confusion matrix revealed that the filter worked well enough to exclude the identified navigation events from further analyses (see Tables S1 and S2).

**Sensitivity Analyses**

Fixing the item difficulties in the disengaged responding class to the inverted number of response options as defined in Equation (3) implies that item responses in the disengaged responding class are indeed randomly guessed, without any regard for the item content. However, previous studies suggest that this is not the case (e.g., Ulitszch et al., 2021). Furthermore, a closer look at the results of both the baseline and the extended model created doubt whether the probability of a correct response really corresponded to chance level. Tables S3 and S4 display the distributions of response options along with the probability of belonging to the solution behavior class for each item for the solution behavior or disengaged responding class, respectively. It can be seen that for most items, the item responses in the disengaged responding class were not evenly distributed between the four response options, as should be expected if disengaged participants really randomly guessed on the items. Therefore, we additionally estimated the extended model with only an equality constraint on the item difficulties in the disengaged responding class. Although the information criteria slightly favored the model with the freely estimated item difficulties, the results did not differ significantly between the models (see table S5). Thus, we assume the results of the extended model to be robust.

| S1  *Number of Indicator Events with and without Filter* | | | |
| --- | --- | --- | --- |
| Indicator | Navigation filter | | |
|  | Without filter | With filter | Percentage deleted |
| Answer change | 5082 | 5081 | < 0.1 % |
| Text reread | 30182 | 30133 | 0.2 % |
| Item revisit | 11720 | 1890 | 83.9 % |

| S2  *Confusion Matrix (N = 500 page visits)* | | |
| --- | --- | --- |
| Own classification | Programmed classification | |
|  | filter | do not filter |
| filter | 37 | 3 |
| do not filter | 10 | 450 |

S3

*Descriptive Results by Item for the Solution Behavior Class Based on Most Likely Class Membership*

| Item | *N* | Percentage of correct responses | Item difficulty | Response time (in seconds) | |  | Final selected response option (in %) | | | |
| --- | --- | --- | --- | --- | --- | --- | --- | --- | --- | --- |
|  |  |  |  | *M* | *SD* |  | A^1^ | B^1^ | C^1^ | D^1^ |
| 1 | 1772 | 75.5 | –1.159 | 31.67 | 28.83 |  | **75.5** | 3.8 | 2.9 | 17.9 |
| 2 | 1708 | 70.7 | –0.880 | 38.87 | 18.01 |  | 11.5 | 12.4 | **70.7** | 5.4 |
| 3 | 840 | 54.4 | 0.218 | 39.72 | 16.05 |  | 17.3 | 27.1 | **54.4** | 1.2 |
| 4 | 1860 | 85.2 | –1.885 | 26.49 | 18.18 |  | **85.2** | 2.1 | 1.7 | 11.0 |
| 5 | 1833 | 50.6 | –0.001 | 22.26 | 12.69 |  | 13.1 | **50.6** | 33.5 | 2.8 |
| 6 | 1816 | 55.4 | –0.217 | 28.37 | 14.27 |  | 23.0 | 7.7 | **55.4** | 13.9 |
| 7 | 1283 | 65.1 | –0.404 | 25.22 | 13.14 |  | 4.8 | **65.1** | 18.4 | 11.7 |
| 8 | 1796 | 38.1 | 0.548 | 30.69 | 17.20 |  | 26.0 | 26.3 | **38.1** | 9.6 |
| 9 | 1787 | 72.8 | –1.060 | 34.08 | 16.88 |  | 3.3 | **72.8** | 15.3 | 8.6 |
| 10 | 1745 | 39.5 | 0.483 | 28.20 | 13.16 |  | 21.4 | **39.5** | 23.6 | 15.5 |
| 11 | 1241 | 63.3 | –0.370 | 37.66 | 17.39 |  | 33.3 | **63.3** | 0.9 | 2.6 |
| 12 | 1531 | 65.9 | –0.695 | 34.21 | 16.80 |  | **65.9** | 15.0 | 11.0 | 8.1 |
| 13 | 1371 | 30.8 | 0.907 | 23.47 | 11.86 |  | 22.9 | 25.9 | 20.4 | **30.8** |
| 14 | 1345 | 71.5 | –0.990 | 18.07 | 12.10 |  | 6.8 | 16.8 | **71.5** | 4.8 |

*Note*. ^1^The correct response is highlighted in bold.

S4

*Descriptive Results by Item for the Disengaged Responding Class Based on Most Likely Class Membership*

| Item | *N* | Percentage of correct responses | Item difficulty | Processing time (in seconds) | |  | Final selected response option (in %) | | | |
| --- | --- | --- | --- | --- | --- | --- | --- | --- | --- | --- |
|  |  |  |  | *M* | *SD* |  | A^1^ | B^1^ | C^1^ | D^1^ |
| 1 | 158 | 17.7 | 1.099 | 13.54 | 4.10 |  | **17.7** | 20.3 | 10.1 | 51.9 |
| 2 | 220 | 19.5 | 1.099 | 15.88 | 6.18 |  | 42.3 | 25.5 | **19.5** | 12.7 |
| 3 | 1088 | 16.3 | 1.099 | 18.69 | 6.98 |  | 42.5 | 40.3 | **16.3** | 0.9 |
| 4 | 37 | 16.2 | 1.099 | 4.30 | 1.89 |  | **16.2** | 48.6 | 27.0 | 8.1 |
| 5 | 66 | 30.3 | 1.099 | 5.29 | 2.63 |  | 6.1 | **30.3** | 51.5 | 12.1 |
| 6 | 53 | 11.3 | 1.099 | 4.57 | 2.00 |  | 24.5 | 56.6 | **11.3** | 7.5 |
| 7 | 584 | 17 | 1.099 | 11.85 | 4.19 |  | 8.2 | **17.0** | 38.4 | 36.5 |
| 8 | 60 | 41.7 | 1.099 | 3.37 | 2.43 |  | 16.7 | 25.0 | **41.7** | 16.7 |
| 9 | 62 | 24.2 | 1.099 | 2.99 | 1.56 |  | 22.6 | **24.2** | 29.0 | 24.2 |
| 10 | 63 | 38.1 | 1.099 | 3.82 | 1.82 |  | 15.9 | **38.1** | 28.6 | 17.5 |
| 11 | 487 | 21.8 | 1.099 | 18.24 | 8.09 |  | 66.7 | **21.8** | 8.4 | 3.1 |
| 12 | 86 | 23.3 | 1.099 | 3.64 | 1.58 |  | **23.3** | 29.1 | 33.7 | 14.0 |
| 13 | 82 | 12.2 | 1.099 | 4.57 | 1.80 |  | 29.3 | 36.6 | 22.0 | **12.2** |
| 14 | 68 | 38.2 | 1.099 | 3.04 | 1.12 |  | 4.4 | 42.6 | **38.2** | 14.7 |

*Note*. ^1^The correct response is highlighted in bold.

| S5  *Results of the Hierarchical Linear Regression for the Model with Equal, but Not Fixed Item Difficulties in the Disengaged Responding Class* | | | | |
| --- | --- | --- | --- | --- |
| Parameter | Estimate | Standard error | *t* | *p* |
| Within level |  |  |  |  |
| General intercept | –20.31 | 7.39 | –2.75 | .006 |
| Intercept item 1 | –6.59 | 2.58 | –2.56 | .011 |
| Intercept item 2 | –10.14 | 3.81 | –2.66 | .008 |
| Intercept item 3 | –13.95 | 4.84 | –2.88 | .004 |
| Intercept item 7 | –8.65 | 3.12 | –2.74 | .006 |
| Intercept item 11 | –13.13 | 4.67 | –2.81 | .001 |
| Intercept item 14 | 4.34 | 3.17 | 1.37 | .172 |
| Item response time | 11.01 | 3.91 | 2.81 | .005 |
| Answer change | –0.73 | 0.78 | –0.94 | .348 |
| Text reread | –2.55 | 1.10 | –2.32 | .020 |
| Item revisit | –1.25 | 0.99 | –1.26 | .206 |
| Between level† |  |  |  |  |
| Variance of $\psi_{i}$ | 8.03 | 7.10 | 1.13 | .258 |
| Covariance between $\psi_{i}$ and $\theta_{i}$ | 2.71 | 0.97 | 2.80 | .005 |
| Correlation between $\psi_{i}$ and $\theta_{i}$ | 0.96 | 0.15 | 6.61 | < .001 |
| *Note.* $\psi_{i}$ defines the individual threshold for response engagement and $\theta_{i}$ the individual reading proficiency of person $i$.  † The variance of $\theta_{i}$ was fixed to 1 for identification. | | | | |
